# *Yifei sanjie* Pills Alleviate Chemotherapy-Related Fatigue by Reducing Skeletal Muscle Injury and Inhibiting Tumor Growth in Lung Cancer Mice

**DOI:** 10.1155/2022/2357616

**Published:** 2022-08-22

**Authors:** Yingchao Wu, Dajin Pi, Yiliu Chen, Qian Zuo, Lizhu Lin, Mingzi Ouyang

**Affiliations:** ^1^School of Traditional Chinese Medicine, Jinan University, Guangzhou 510632, Guangdong, China; ^2^MOE Key Laboratory of Tumor Molecular Biology and Key Laboratory of Functional Protein Research of Guangdong Higher Education Institutes, Institute of Life and Health Engineering, College of Life Science and Technology, Jinan University, Guangzhou 510632, Guangdong, China; ^3^Oncology Center, The First Affiliated Hospital of Guangzhou University of Chinese Medicine, Guangzhou 510405, Guangdong, China

## Abstract

Chemotherapy-related fatigue (CRF), one of the most severe adverse effects observed in cancer patients, has been theoretically related to oxidative stress, and antioxidant treatment might be one of the most valuable therapeutic approaches. However, there are still few effective pharmacological therapies. *Yifei Sanjie pills* (YFSJ), a classical formula used to treat lung cancer as complementary and alternative medicine, have been proved to alleviate CRF of lung cancer patients in clinical practices. However, the underlying mechanisms have not been clarified. In this study, our data showed that YFSJ alleviated CRF presented as reversing the decline of swimming time and locomotor activity induced by cisplatin (DDP). Moreover, YFSJ significantly reduces the accidence of mitophagy and mitochondrial damage and reduces apoptosis in skeletal muscle tissues caused by DDP. It probably works by decreasing the oxidative stress, inhibiting the activation of the AMPK/mTOR pathway, decreasing protein expression levels of Beclin1 and other autophagy-related proteins, and attenuating the activation of Cytochrome c (cyto. C), Cleaved Caspase-9 (c-Casp 9), and other apoptosis-related proteins. Furthermore, YFSJ enhanced DDP sensitivity by specifically promoting oxidative stress and activating apoptosis and autophagy in the tumor tissues of mice. It was also found that YFSJ reduced the loss of body weight caused by DDP, reversed the ascent of serum concentrations of alanine aminotransferase (ALT), aminotransferase (AST), and creatinine (CREA), increased the spleen index, and prolonged the survival time of mice. Taken together, these results revealed that YFSJ could alleviate CRF by reducing mitophagy and apoptosis induced by oxidative stress in skeletal muscle; these results also displayed the effects of YFSJ on enhancing chemotherapy sensitivity, improving quality of life, and prolonging survival time in lung cancer mice received DDP chemotherapy.

## 1. Introduction

Although advances have been made in targeted therapy and immunotherapy, platinum-based chemotherapy is still the standard treatment for advanced lung cancer, with a large proportion [1–3]. Chemotherapy-related fatigue (CRF), one of the most severe adverse effects caused by chemotherapeutic treatments, has been observed in 27–82% of patients with advanced lung cancer [4], presenting as a persistent distressing, subjective sense of tiredness or exhaustion [5], which affects the long-term quality of life or even result in the deaths of patients [6].

Despite the prevalence of this condition, the etiology of CRF has not been fully elucidated. Some studies found that the possible mechanisms were associated with energy unbalance, inflammation, changes in circadian rhythm, depression, and immune system disorders [7]. However, increasing evidence indicates that the occurrence of CRF is related to the dysfunction of skeletal muscle, which has an essential proportion in maintaining the energy homeostasis of the human body [8]. A study on chemotherapy-treated patients reported that chemotherapy always indistinguishably targets the mitochondria of both cancerous and noncancerous cells, inducing fatigue due to high oxidative stress [9–12]. Its potential mechanism is that the damage pathways of noncancerous cells, especially in highly metabolic organs such as skeletal muscle, will be stimulated by adaptive responses to oxidative stress and induce reactive oxygen species (ROS) overgeneration [13–18]. Accumulation of ROS-caused mitochondrial damage can be removed by an autophagic process called “mitophagy” to maintain cell homeostasis [19]. However, when more devastating damage is beyond the capability of mitophagy, the dysfunctional mitochondria will induce apoptotic cell death, which can lead to the onset of CRF [20, 21]. While CRF has been theoretically related to oxidative stress, and antioxidant treatment might be one of the most valuable therapeutic approaches for CRF, there are still few effective pharmacological therapies that can successfully eliminate CRF [22]. At the same time, tumor size is also closely related to CRF [23]. Therefore, further research is urgently needed to raise new interventions to prevent, postpone or eliminate fatigue in cancer patients.

The use of Traditional Chinese Medicine (TCM) might be one such possible strategy, which is wildly used in CRF treatment to reduce fatigue or improve the quality of life in cancer patients [24]. *Yifei Sanjie pills* (YFSJ), also known as “*Yiqi Chutan Tang*,” is a TCM formula used to treat lung cancer as complementary and alternative medicine, which consists of *Panax quinquefolius Radix* (Xi yang shen), *Ranunculi Ternati Radix* (Mao zhao cao), *Sarcandrae Herba* (Zhong jie feng), *Pinelliae Rhizoma Praeparatum* (Fa ban xia), *Ganoderma* (Ling zhi), *Bombyx batryticatus* (Chao jiang can), *Cremastrae Pseudobulbus Pleiones Pseudobulbus* (Shan ci gu), and *Fritillariae Thunbergii Bulbus* (Zhe bei mu). Previous studies reported that the major herbs of YFSJ, such as *Panax quinquefolius Radix* and *Ganoderma,* have been approved for anti-CRF effects in the clinic and preclinic studies due to the inhibition of oxidative stress and the improvement of mitochondrial function in skeletal muscles [25–32]. Phytochemicals absorbed from *Thunbergii Bulbus*, *Bombyx batryticatus,* and *Pinelliae Rhizoma Praeparatum* could also reduce oxidative stress by decreasing free radical formation and scavenging free radicals [33–35], which were commonly associated with fatigue. Furthermore, our previous clinical practices have shown the effects of YFSJ to prolong the median survival time [36, 37] and alleviate CRF in non-small-cell lung cancer (NSCLC) patients [38, 39]. However, the underlying mechanisms have not been clarified. In this study, in addition to its usual antitumor effects, we have focused on the oxidative stress, mitochondria autophagy, and apoptosis in skeletal muscle of CRF mice model to demonstrate the underlying mechanisms of YFSJ against CRF.

## 2. Materials and Methods

### 2.1. YFSJ Preparation

YFSJ was composed of eight herbs formed by pill preparation and was purchased from the First Affiliated Hospital of Guangzhou University of Chinese Medicine (Guangdong, China). YFSJ (8 g/packet) was dissolved in 24 mL normal saline, and the solution was promoted by eddy vibration to the final concentration of 0.33 g/mL before use. Details of the herbal materials are listed in Supplementary Figure 1 and Supplementary Table 1. Chemical constituents of YFSJ were identified based on the Q-Orbitrap high-resolution liquid/mass spectrometry (Q-Orbitrap-LC/MS). The data collected by high-resolution liquid mass were processed by CD2.1 (Thermo Fisher) and then searched and compared in the database (MZCloud, MZVault, ChemSpider). The peak intensity chromatograms of chemical constituents in YFSJ were displayed in Supplementary Figure 2. The obtained compounds were cross-linked with the known traditional Chinese medicine components in YFSJ to screen out the possible compounds listed in Supplementary Table 2.

### 2.2. Chemicals and Reagents

Cisplatin (DDP, Cat.^#^H20010743) injection was purchased from Jiangsu Hausen Pharmaceutical Co., Ltd (Jiangsu, China). Dulbecco's modified Eagle's medium (DMEM, Cat.^#^11965092), fetal bovine serum (FBS, Cat.^#^10270106), penicillin/streptomycin (Cat.^#^10378016), and phosphate-buffered saline (PBS, Cat.^#^10010023) were supplied by Gibco (NY, USA). Primary antibodies against Beclin1 (Cat.^#^11306-1-AP), p62 (Cat.^#^66184-1-Ig), Cytochrome *c* (cyto. C, Cat.^#^66264-1-Ig), and DAPK1 (Cat.^#^25136-1-AP) were purchased from Proteintech (Wuhan, China). Primary antibodies against Phospho-AMPK*α* (p-AMPK*α*, Cat.^#^50081S), Phospho-mTOR (p-mTOR, Cat.^#^5536T), Atg7 (Cat.^#^8558T), LC3A/B (Cat.^#^12741S), Cleaved Caspase-9 (c-Casp 9, Cat.^#^20750S), Cleaved Caspase-3 (c-Casp 3, Cat.^#^9661T), Cleaved PARP (c-PARP, Cat.^#^5625T), Phospho-SAPK/JNK (p-JNK, Cat.^#^9255S), Phospho-p53 (p-p53, Cat.^#^9284T), Bax (Cat.^#^2772T), and GAPDH (Cat.^#^5174T) and rabbit (Cat.^#^7074P2) or mouse (Cat.^#^7076P2) secondary antibodies were purchased from Cell Signaling Technology (Danvers, MA, USA).

### 2.3. Cell Culture

Lewis lung cancer (LLC) cells were acquired from the Guangzhou University of Chinese Medicine (Guangzhou, China). The cells were cultured in the DMEM medium containing 10% FBS, 1% streptomycin, and 1% penicillin and maintained in a 5% CO_2_ incubator at 37°C. Cells were subcultured when the density reached 90% to proliferate enough and were used for subsequent experiments.

### 2.4. Lung Cancer Xenogeneic Mouse Model

A total of 100 five-week-old C57/BL mice (15 ± 1 g) were purchased from Beijing HFK Bioscience Co., Ltd., [Approval No. SCXK (Jing) 2019–0008, Beijing, China]. All experiments were conducted according to the relevant laws and institutional guidelines and with the approval of the Animal Ethics Committee of Jinan University (Approval No. IACUC-20200923-06). After acclimatizing for 10 days, 20 mice were randomly selected as the NC group (normal control). The remaining mice were given subcutaneous injections with 1 × 10^6^ LLC cells in the right flank to establish the lung cancer xenogeneic mice model. When the tumor size reached 80–100 mm^3^, the xenografts mice were randomly divided into four groups (*n* = 20): (1) TC group (mice with tumor but without any treatment); (2) DDP group (mice with tumor and treated with DDP, intraperitoneal, 5 mg/kg, 0.1 ml, once weekly); (3) DDP + YFSJ group (mice with tumor and cotreated with DDP and YFSJ; DDP, intraperitoneal, 5 mg/kg, 0.1 ml, once weekly; YFSJ, intragastrical, 3 g/kg, 0.2 ml, once daily); (4) YFSJ group (mice with tumor and treated with YFSJ, intragastrical, 3 g/kg, 0.2 ml, once daily). The NC and TC groups have received administration of normal saline (with the same route and volume of DDP and YFSJ). Each group of mice was randomly divided into two subgroups, one for survival analysis and the other for other experiments.

### 2.5. Behavioral Tests

#### 2.5.1. Weight-Loaded Swimming Test (WST)

Mice were subjected to WST before administration and on the 7th, 14th, and 21st days after administration, respectively, as the previous study described [29]. The mouse was placed separately in the swimming pool with 20 cm in diameter, 35 cm high, and at stationary temperature (25°C ± 1°C), where the mouse could just touch the bottom with its feet to support itself. A 7% of the body weight tin wire was fixed on the root of the tail to weight load the mouse. When it failed to rise to the water surface to breathe within a 10 sec period, the mouse would be determined to be exhausted and salvaged from the water, dried with a towel, and placed back in the original cage. The time spent by the mouse floating in the water with necessary movements until exhausting its strength was recorded, which was considered a negative correlation with fatigue.

#### 2.5.2. Open-Field Test (OFT)

Twenty-four hours after the WST, the OFT was conducted in an arena made of plexiglass (100 × 100 × 50 cm^3^). The arena was divided into the center area with a 50 × 50 cm^2^ square and the peripheral area with twelve 25 × 25 cm^2^ squares. The mouse was placed separately in the arena for 5 minutes. Locomotor activity was monitored using an infrared camera. The total movement distance, distance, and time spent in each mouse's central and peripheral areas in the specified observation time were calculated and analyzed using the EthoVision XT 14 software (Noldus Information Technology Co., Ltd, Beijing, China). Each mouse was returned to its home cage after the behavioral test. The OFT apparatus was thoroughly cleaned with 70% ethyl alcohol to eliminate any olfactory cues between tests.

### 2.6. Determination of the Survival Time of Mice, Body Weight, and Tumor Volume of YFSJ

Ten mice from each group were randomly selected for survival analysis, and the survival time was recorded up to 90 days after the beginning of treatment. At the end of the survival analysis, the still-alive mice were terminated individually by deeply anesthetized with pentobarbital sodium by intraperitoneal injection (150 mg/kg). Another ten mice from each group were selected for other experiments, and the body weight and tumor volume of the mice were measured every three days. The computational formula of tumor volume = long diameter (*L*) × short diameter (*W*)^2^ × 0.5.

### 2.7. Mouse Sacrifice, Sample Collection, and Detection of Serum Biochemical Indices

After all behavioral tests, the other 10 mice of each group were anesthetized with isoflurane inhalation (RWD Life Science Pharmaceutical Co., Ltd., Shenzhen, China). The tumors and bilateral gastrocnemius muscles of mice were immediately removed on ice for the following examination when the mice were unconscious. The spleen was removed and weighed to measure the spleen index (weight of spleen/body weight × 10 × 100%). The whole blood samples were collected, placed at room temperature for 2 hours, and centrifuged at 3000 r/min at 4°C for 15 min, and the supernatants were taken for biochemical measurement. The levels of serous alanine aminotransferase (ALT), aminotransferase (AST), and creatinine (CREA) were detected according to the procedures provided in the kits (Rayto Life Sciences Inc., Shenzhen, China) and analyzed by the automatic biochemical analyzer Chemray 800 (Redu Life Technology Inc., Shengzhen, Chian).

### 2.8. Determination of the Concentrations of Oxidative Stress Markers

The tissue ROS assay kit (Cat.^#^BB-470532, BestBio, Shanghai, China) was used to measure gastrocnemius muscle and xenograft tumor tissues ROS concentrations. The Cu/Zn-SOD and Mn-SOD assay kit with WST-8 (Cat.^#^S0103, Beyotime, Shanghai, China) was used to measure gastrocnemius muscle and xenograft tumor tissues SOD concentrations. The Lipid Peroxidation MDA assay kit (Cat.^#^S0131S, Beyotime, Shanghai, China) was used to measure MDA concentrations in gastrocnemius muscle and xenograft tumor tissues. In short, we completed the determination of ROS, SOD, and MDA concentrations according to the kit's instructions. These experiments were performed in triplicate. ROS data are expressed as the percentage of the fluorescence intensity relative to that of the control group. The data of SOD are expressed in U/mg protein. The data of MDA are expressed in *μ*mol/mg protein.

### 2.9. Examination of the Gastrocnemius Muscle Tissues

#### 2.9.1. Electron Microscopy (EM)

The fresh muscle tissue was dissected further into 2 × 2 mm^3^ samples immediately, fixed with 2.5% glutaraldehyde for 4 h at 4°C, and triple rinsed with PBS. Then, the samples were fixed with 1% osmic acid for 2 h, triple rinsed with PBS again, and dehydrated with alcohol of gradient concentration (50%, 70%, 80%, 90%, and 95%) acetone for 20 min. After that, muscle tissue was immersed in Epon812 overnight and polymerized in a 45°C drying oven for 12 h. At last, samples were cut into 70 nm slices, stained with uranyl acetate and lead citrate, and captured under an electron microscope (HITACHI HT7700, Japan). The condition of mitochondria and autophagosomes was assessed using Image-pro plus 6.0 system (Media Cybernetics, Inc., Rockville, USA).

#### 2.9.2. Hematoxylin and Eosin (HE) Staining

A portion of the left fresh muscle tissue was fixed with 4% paraformaldehyde, dehydrated, embedded in paraffin, cut into 5 *μ*m slices, then stained with hematoxylin for 30 min and eosin for 5 min, vitrified with xylene, and sealed with neutral resin. The stained slices were observed and photographed under a light microscope at 200x magnification (NIKON Eclipse ci, Japan). Semiquantitative scoring of tissue lesions was calculated according to El-Far et al. [40]. Briefly, lesions in 3 fields were chosen randomly from each slide for each mouse and averaged. The lesions were scored in a blinded way (score scale: 0 = normal; 1 ≤ 25%; 2 = 26–50%; 3 = 51–75%; and 4 = 76–100%).

#### 2.9.3. Terminal-Deoxynucleoitidyl Transferase Mediated Nick End Labeling (TUNEL) Staining

The rest paraffin-embedded muscle tissue section detected the apoptosis level using the TUNEL apoptosis detection kit (AtaGenix, Hubei, China). After dewaxing, the section was added 20 *μ*g/mL protease K for 30 min at 37°C and triple rinsed in PBS. Then, the samples were stained with 4′, 6-diamidino-2-phenylindole (DAPI, 0.05 *μ*g/mL, Servicebio, Wuhan, China) in PBS for 10 min and sealed after rinsing three times with PBS. Fluorescent images were captured at 400x magnification under a fluorescence microscope (NIKON Eclipse ci, Japan). These experiments were performed in triplicate. The integrated densities of TUNEL-positive areas were measured using ImageJ (v1.46r; NIH, Bethesda, MD, USA) [41].

### 2.10. Western Blotting

An appropriate amount of mouse's tumor and right gastrocnemius muscle were dissected and immediately separated on ice. Tissues were homogenized with the Scientz-48 High-throughput tissue grinder (Xinzhi Biological Co., Ltd, Ningbo, China) and RIPA Lysis Buffer (Cell Signaling Technology, Inc. Massachusetts, USA), centrifuged at 4°C, 12000*g* for 10 min. The supernatant was collected for detecting protein concentration and western blot analysis. The protein concentration in the obtained supernatant was determined using an Enhanced BCA Protein Assay Kit (Beyotime Biotechnology, Shanghai, China). The target protein was separated with 8–10% SDS-PAGE (Beyotime, Shanghai), transferred to a polyvinylidene fluoride (PVDF) membrane (Millipore, Marlborough, USA), sealed with 5% skimmed milk powder at room temperature for 60 min, and incubated with the primary antibodies at 4°C overnight: anti-p-AMPK*α* (1 : 1000), anti-p-mTOR (1 : 1000), anti-Beclin1 (1 : 1000), anti-Atg7 (1 : 1000), anti-LC3A/B (1 : 1000), anti-p62 (1 : 1000), anti-cyto. C (1 : 1000), anti-c-Casp 9 (1 : 1000), anti-c-Casp 3 (1 : 1000), anti-c-PARP (1 : 1000), anti-p-JNK (1 : 1000), anti-p-p53 (1 : 1000), anti-Bax (1 : 1000), anti-DAPK1 (1 : 1000), and anti-GAPDH (1 : 2500). Subsequently, the target protein was washed with Tris-buﬀered Saline Tween-20 (TBST) solution three times, incubated with the corresponding secondary anti-rabbit or anti-mouse antibody (1 : 5000) at room temperature for 60 min, washed with TBST solution three times again, and visualized by hypersensitive ECL kit (Beyotime, Shanghai). These experiments were performed in triplicate. Density values of the bands were captured and documented through a gel image analysis system (ChemiDox™, Bio-Rad, USA) and normalized to GAPDH.

### 2.11. Statistical Analysis

All data were expressed as the mean ± standard deviation (SD) and analyzed by SPSS 13.0 (SPSS Inc., IL, USA) or GraphPad Prism 9 software (GraphPad Software, LLC, California, USA). The experimental data of repeated observations were analyzed with repeated measures ANOVA. The survival times of animals were analyzed with Kaplan–Meier analysis. The other data were analyzed by one-way ANOVA and Student's *t*-test. The significance of statistical differences was considered at *P* less than 0.05.

## 3. Result

### 3.1. The Effects of YFSJ on CRF in Mice

**Figure 1:**
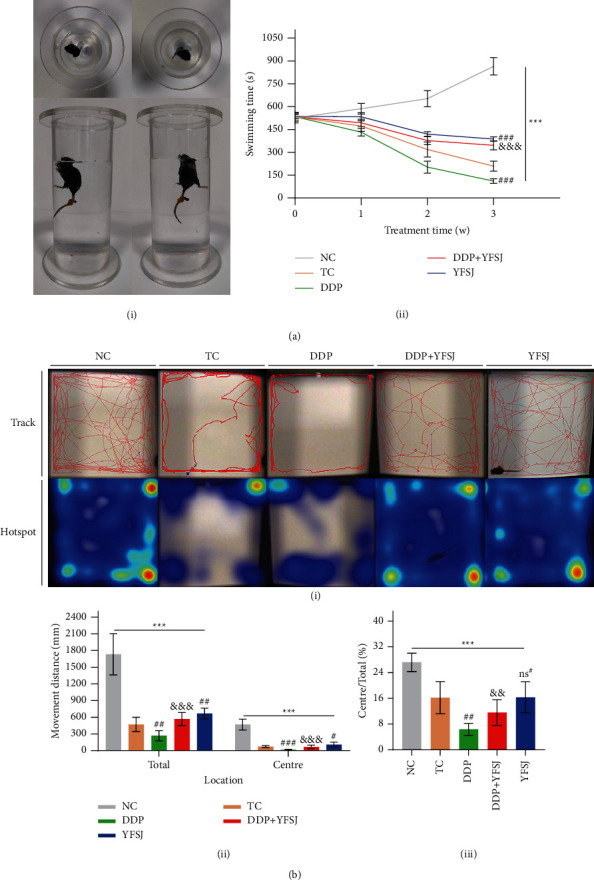
The effects of YFSJ on CRF. (a) WST: (i) schematic diagram of WST; (ii) the swimming time of the WST. (b) OFT: (i) pathway map and regional residence time heatmap of mouse movement in OFT; (ii) the movement distance of mice, including total movement distance and central movement distance; (iii) the proportion of the movement distance in the central region to the total movement distance of mice. The data are presented as the means ± SD of triplicate experiments, *n* = 10. ^ns^^*∗*^*p* > 0.05, ^*∗∗∗*^*p* < 0.001 compared with the NC group. ^#^*p* < 0.05, ^##^*p* < 0.01, ^###^*p* < 0.001 compared with the TC group. ^&&^*p* < 0.01, ^&&&^*p* < 0.001 compared with the DDP group.

To evaluate the effect of YFSJ on CRF in mice, we assessed the swimming time of the WST, total movement distance, and the ratio of the central region to the total movement distance of the OFT. As is shown in Figure 1(a), the tumor-bearing group mice with/without treatment showed a significant decrease in swimming time compared to the mice in the tumor-free group (NC group). Meanwhile, the DDP treatment induced a pronounced decrease in the swimming time of tumor-bearing mice. However, the decline in swimming time induced by DDP was significantly reversed after 21 days of treatment with YFSJ. Additionally, the swimming time of tumor-bearing mice in the YFSJ group is longer than in the TC group, indicating the dual role of YFSJ in treating both cancer- and chemotherapy-related fatigues. It is further validated by the results of movement distance and residence time in the central area that YFSJ treatment not only significantly extended the total movement distance of mice but also increased the ratio of the central region to total movement distance in comparison with mice treated with DDP alone (Figure 1(b)). However, there was no significant change in the ratio of the central region to total movement distance among mice in the YFSJ and TC groups (Figure 1(b) iii). These data collectively demonstrated that YFSJ treatment might play a key role in alleviating CRF.

### 3.2. The Effects of YFSJ on Oxidative Stress-Induced Mitophagy in Mouse Skeletal Muscle

**Figure 2:**
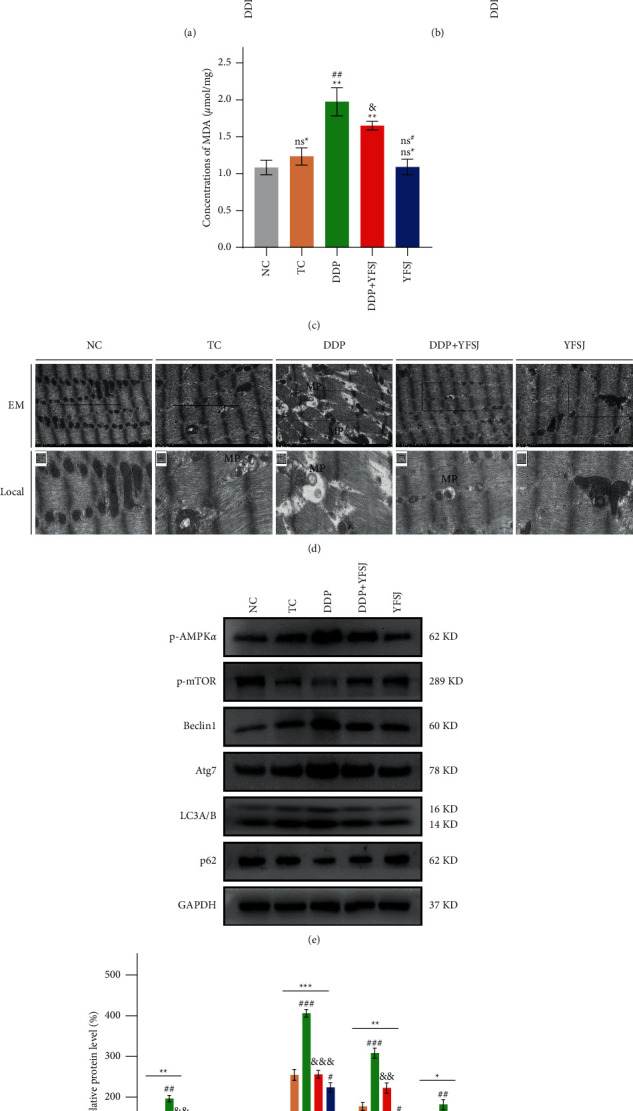
The effects of YFSJ on oxidative stress-induced mitophagy in mouse skeletal muscle. Indicators of oxidative stress in skeletal muscle tissues: (a) the relative concentrations of ROS in skeletal muscle tissues; (b) the SOD concentrations in skeletal muscle tissues; (c) the MDA concentrations in skeletal muscle tissues. (d) Electron microscopy of mitophagy in skeletal muscle tissues. Scale bars = 2 *μ*m; local magnification = 15X; MP = mitophagosomes. Autophagy-related proteins in skeletal muscle tissues: (e) Western blotting bands of autophagy-related proteins; (f) relative expression levels of autophagy-related proteins. The data are presented as the means ± SD of triplicate experiments, *n* = 3. ^ns^^*∗*^*p* > 0.05, ^*∗*^*p* > 0.05, ^*∗∗*^*p* < 0.01, ^*∗∗∗*^*p* < 0.001 compared with the NC group. ^ns#^*p* > 0.05, ^#^*p* < 0.05, ^##^*p* < 0.01, ^###^*p* < 0.001 compared with the TC group. ^&^*p* < 0.05, ^&&^*p* < 0.01, ^&&&^*p* < 0.001 compared with the DDP group.

Since CRF is characterized by increased oxidative stress and dysfunctional mitochondria, which are involved in regulating mitochondria autophagy, we proposed that the restrained oxidative stress by YFSJ treatment contributes to the inhibition of autophagy in skeletal muscle cells; thus, the effect of YFSJ on oxidative stress is assessed. We first examined the ROS, SOD, and MDA concentrations in mice to evaluate the effect of YFSJ on oxidative stresses. These data revealed that the aberrant production of ROS and MDA by DDP treatment could be significantly rescued by YFSJ (Figures 2(a)–2(c)). Consistently, the reduced levels of SOD were markedly elevated in DDP + YFSJ and YFSJ groups (Figure 2(b)). As shown in the electron microscopy of skeletal muscle mitochondria (Figure 2(d)), the DDP group manifested increasing swollen, vacuolar damaged mitochondria, which were surrounded by the double membrane phagophore structure forming autophagosomes compared to the TC group. However, YFSJ significantly inhibited the formation of autophagosomes. Moreover, Western blot data showed that YFSJ treatment inhibited the activation of the AMPK/mTOR pathway, counteracting the accumulated turnover of LC3-A to LC3-B by DDP, which was further evidenced by the changed protein expression levels of Beclin1, Atg7, and p62 in skeletal muscle cells of mice from the DDP + YFSJ group (Figures 2(e) and 2(f)). Taken together, these results revealed that oxidative stress-mediated mitophagy is responsible for the inhibitory effect of YFSJ on CRF.

### 3.3. The Effects of YFSJ on Chemotherapy-Induced Apoptosis in Mouse Skeletal Muscle

**Figure 3:**
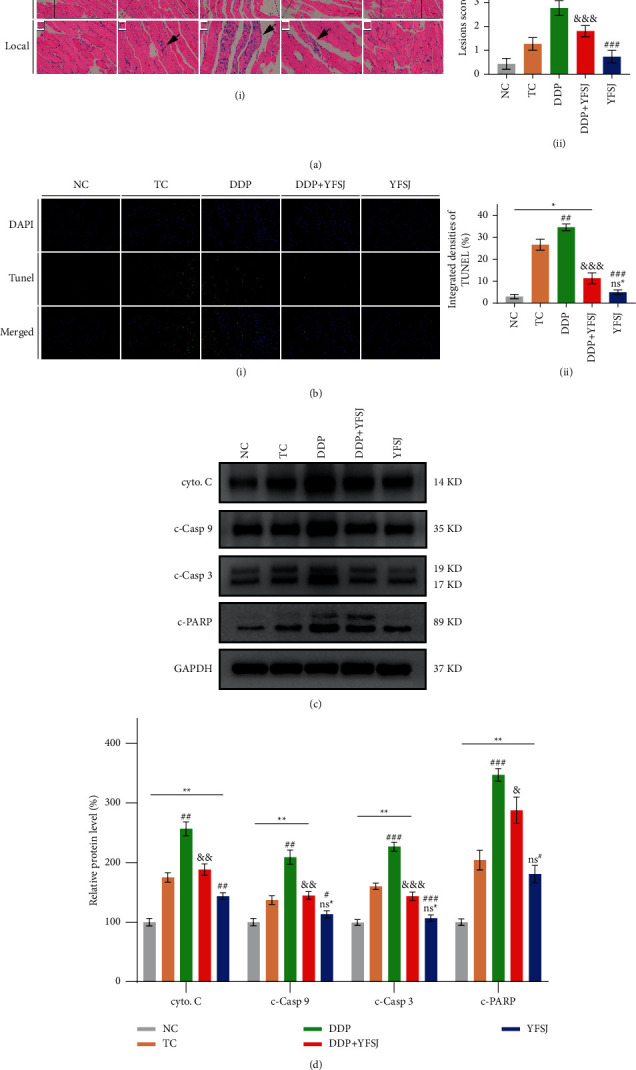
The effects of YFSJ on chemotherapy-induced apoptosis in mouse skeletal muscle. (a) (i) HE staining of skeletal muscle tissues. Scale bars = 200 *μ*m; local magnification = 15x. (ii) HE semiquantitative scoring of lesions (*n* = 10). (b) (i) TUNEL staining of skeletal muscle tissues. Magnification = 100x. (ii) Integrated densities of TUNEL (*n* = 3). Apoptosis-related proteins in skeletal muscle tissues: (c) western blotting bands of apoptosis-related proteins; (d) relative expression levels of apoptosis-related proteins. The data are presented as the means ± SD of triplicate experiments, *n* = 3. ^ns^^*∗*^*p* > 0.05, ^*∗*^*p* < 0.05, ^*∗∗*^*p* < 0.01 compared with the NC group. ^ns#^*p* > 0.05, ^#^*p* < 0.05, ^##^*p* < 0.01, ^###^*p* < 0.001 compared with the TC group. ^&^*p* < 0.05, ^&&^*p* < 0.01, ^&&&^*p* < 0.001 compared with the DDP group.

HE staining results showed that the tissue structures of skeletal muscles were clear and complete in the DDP + YFSJ group while destroyed in the DDP group with an amount of muscle fiber breakage, such as swelling between muscle cells or broken connections between muscle cells (Figure 3(a)). TUNEL assay was performed to detect apoptosis levels. As shown in Figure 3(b), mice treated with DDP + YFSJ attenuated the elevated skeletal muscle cell apoptosis by DDP. To substantiate the above findings, a series of proapoptotic proteins (e.g., cyto. C, c-Casp 9, c-Casp 3, and c-PARP) were detected. Western blot data revealed that YFSJ treatment attenuated the promoting effect of DDP on skeletal muscle cell apoptosis (Figure 3(c)), which is consistent with the results of the TUNEL assay. Thus, reduced apoptosis in skeletal muscle cells by alleviating oxidative-mediated mitophagy may be the potential mechanism of YFSJ in the treatment of CRF.

### 3.4. The Effects of YFSJ on Xenograft Tumors in Mice

**Figure 4:**
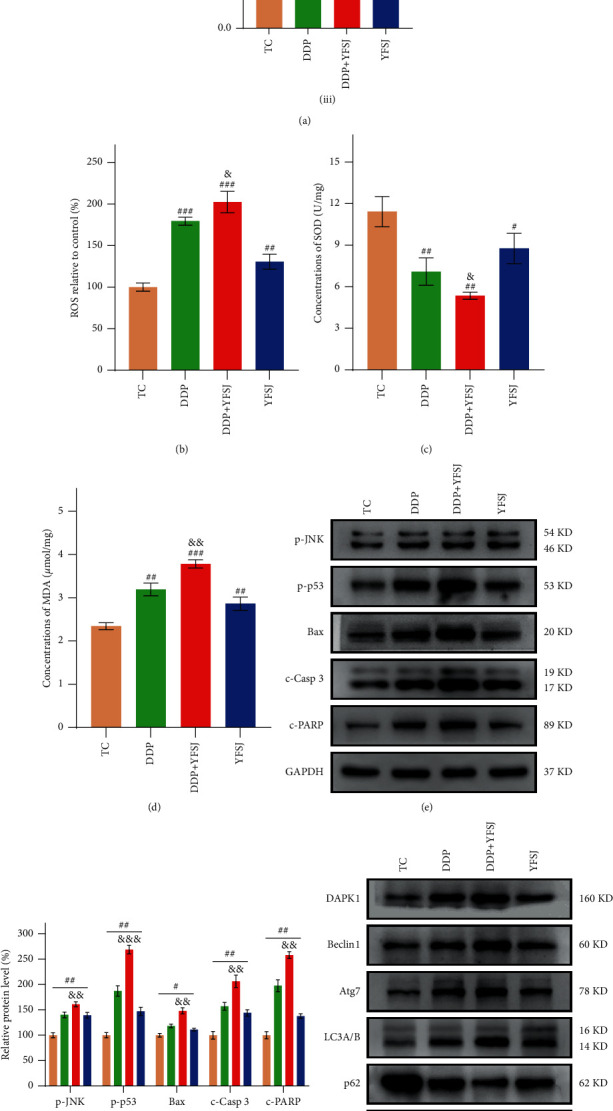
The effects of YFSJ on xenografts in mice. (a) The condition of the tumor: (i). The volume of the tumor, *n* = 10; (ii) representative image pictures of the tumor; (iii) weight of tumor in mice, *n* = 10. Indicators of oxidative stress in tumor tissues: (b) the relative concentrations of ROS in tumor tissues; (c) the SOD concentrations in tumor tissues; (d) the MDA concentrations in tumor tissues. Apoptosis-related proteins in tumor tissues: (e) western blotting bands of apoptosis-related proteins; (f) relative expression levels of apoptosis-related proteins. Autophagy-related proteins in tumor tissues: (g) western blotting bands of autophagy-related proteins; (h) relative expression levels of autophagy-related proteins. The data are presented as the means ± SD of triplicate experiments, *n* = 3, unless otherwise specified. ^#^*p* < 0.05, ^##^*p* < 0.01, ^###^*p* < 0.001 compared with the TC group. ^&&^*p* < 0.01, ^&&&^*p* < 0.001 compared with the DDP group.

The antitumor effects of YFSJ were investigated in vivo. As is shown in Figure 4(a), xenografts-bearing mice treated with DDP + YFSJ exhibited the lowest growth rate compared to other groups. Tumor size and tumor weight were significantly reduced in DDP + YFSJ-treated mice. Although xenografts treated with DDP or YFSJ alone showed a decrease compared to xenografts in the TC group, the combined treatment of DDP and YFSJ exhibited a more significant antitumor effect. Additionally, we assessed the ROS, SOD, and MDA concentrations in tumor tissues. The results demonstrated that either single or combined treatment could significantly promote ROS and MDA production (Figures 4(b) and 4(d)), while SOD, as an antioxidant index, showed an opposite trend (Figure 4(c)). Subsequently, Western blots were performed to detect apoptosis and autophagy in xenografts. Consistently, xenografts treated with DDP or YFSJ alone both displayed activated cell apoptosis and autophagy, while the combined treatment of DDP + YFSJ showed the most potent effects (Figures 4(e)–4(h)), as evidenced by activation of the JNK/p53 pathway, increasing Bax, c-Casp 3, c-PARP, Beclin1, Atg7, and LC3B and reducing p62 in protein expression levels. In conclusion, YFSJ has an inhibitory effect on tumor growth and is synergistic with the antitumor effect of DDP.

### 3.5. The Effects of YFSJ on the Quality of Life and Survival Time in Mice

**Figure 5:**
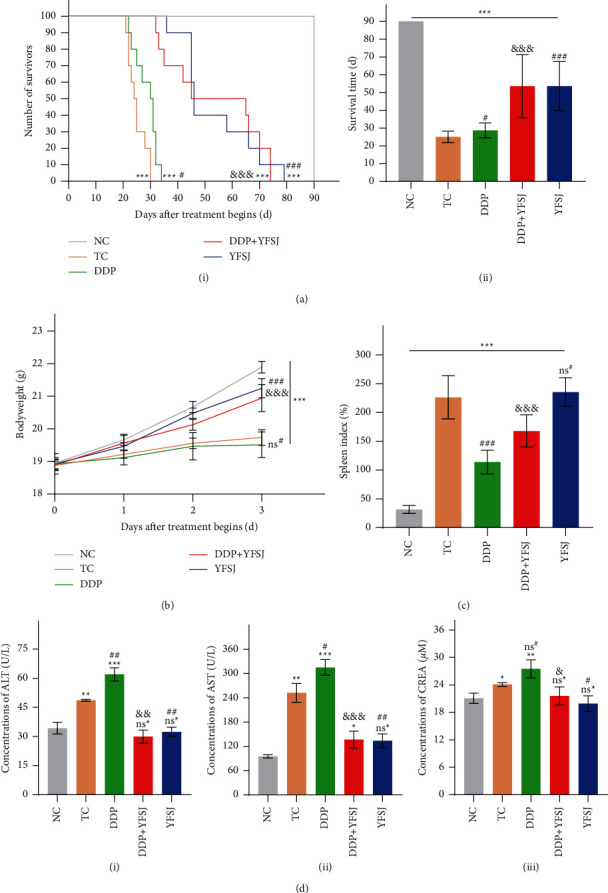
The effects of YFSJ on the quality of life and survival time in mice. (a) Survival analysis: (i) survival curve in mice; (ii) Mouse survival time. (b) The body weight of mice. (c) Spleen index in mice. (d) Serum biochemical indices of mice, *n* = 3: (i) the ALT concentrations in mice serum; (ii) the AST concentrations in mice serum; (iii) the CREA concentrations in mice serum. The data are presented as the means ± SD of triplicate experiments, *n* = 10. ^ns^^*∗*^*p* > 0.05, ^*∗*^*p* < 0.05, ^*∗∗*^*p* < 0.01, ^*∗∗∗*^*p* < 0.001 compared with the NC group. ^ns#^p > 0.05, ^#^*p* < 0.05, ^##^*p* < 0.01, ^###^*p* < 0.001 compared with the TC group. ^&^*p* < 0.05, ^&&^*p* < 0.01, ^&&&^*p* < 0.001 compared with the DDP group.

Accumulating evidence has highlighted the importance of CRF in patients with cancers' quality of life and survival time [42]. To investigate whether reduced fatigue of mice by YFSJ treatment could contribute to a better prognosis and desirable quality of life, we evaluated the survival time, body weight, and serum concentrations of ALT, AST, and CREA of the mice. As is shown in Figure 5(a), the survival time of mice in the NC group is the longest compared to tumor-bearing mice in other groups. Further analysis revealed that DDP treatment did not substantially prolong the survival time. In contrast, the combined therapies of DDP and YFSJ or YFSJ treatment alone could significantly extend the survival time of tumor-bearing mice. Afterward, body weight, ALT, AST, and CREA concentrations were detected. The results demonstrated that compared with the TC group, mice's body weights were slightly reduced after DDP treatments. Nevertheless, YFSJ treatments significantly restore the body weight loss of mice induced by DDP (Figure 5(b)). Analysis of serum markers exhibited ascending concentrations of ALT, AST, and CREA in mice of the DDP group while descending levels in the DDP + YFSJ group (Figure 5(d)). Pathologically, the spleen was compensatively enlarged due to the retention of immunocytes in the spleen [43]. As a result, the spleen index of mice in the TC group was the highest. By contrast, the spleen index of the DDP-treated group was reduced significantly; the DDP + YFSJ group increased the spleen index compared to mice treated with DDP alone, which indicated the immunosuppression resulting from the DDP treatment could be activated by YFSJ (Figure 5(c)). The above results revealed that YFSJ could overcome the toxicity and improve the quality of life of mice by DDP.

## 4. Discussion

YFSJ is a proprietary medicine developed from traditional Chinese medicine prescriptions, Yiqi Chutan Tang, with lung cancer treatment. Interestingly, in clinical observation, we found that it had a significant effect on the improvement of CRF [38, 39]. In order to clarify the underlying mechanisms, in the current research, the xenogeneic model was established in C57/BL mice using LLC cells and treated with DDP to simulate a CRF situation that occurs in humans; the WST and OFT were used for the evaluation of fatigue related to chemotherapy. Moreover, we used the model to evaluate the effect of YFSJ on the fatigue caused by DDP.

The WST assessed the endurance and fatigue status [44]. The indices of locomotor activities were tested in the OFT [45–47] to evaluate the physical and psychical fatigue, respectively. In this study, the TC and DDP group showed more obvious physical and psychical fatigue than the NC group by significantly decreasing swimming time, less locomotor distance, and residence time in the central area. Between the two groups, the DDP group presented more severe fatigue than the TC group, reflecting that the fatigue of tumor-bearing mice was aggravated by DDP chemotherapy, which was accordant with previous research that some cancer patients presented severer fatigue after receiving chemotherapy [48, 49]. At the same time, the decline in swimming time and locomotor activities induced by DDP were significantly reversed after 21 days of treatment with YFSJ, indicating that YFSJ could alleviate the CRF, which was consistent with previous clinical studies [13–15, 38, 39, 50].

Numerous studies proved that increased oxidative stress would aggravate muscle fatigue [51–53]. Oxidative stress can be produced by tumors themselves [54] and directly or indirectly given rise by numerous chemotherapeutic agents [55, 56]. In the oxidative stress state, overproduced free radicals like ROS will ultimately produce MDA, directly reflecting the degree of lipid peroxidation [57]. An antioxidant such as SOD plays an essential role in removing these productions of oxidative stress [58]. Therefore, ROS, SOD, and MDA were chosen as biomarkers to evaluate the degree of oxidative stress in skeletal muscle. This study showed that the DDP elevated the ROS and MDA concentrations and weakened the SOD activity significantly in the muscle tissue; however, the oxidative stress phenomena were alleviated significantly in the DDP + YFSJ group. That may be related to some components of YFSJ (Supplementary Table 2), such as ginsenoside Rg2 and ginsenoside Rg3 [59], which have been shown to have antioxidant effects on normal tissues. At the same time, oxidative stress-induced damage to mitochondrial DNA, membrane lipids, and proteins can be degraded by mitophagy [60]. That is because oxidative stress leads to phosphorylation of AMPK*α* in the mitochondria, which activates AMPK [61]. Activation of AMPK inhibited the phosphorylation of mTOR [62] and weakened the inhibition of p-mTOR on the autophagy key factor Beclin1 [63]. Mechanistically, Beclin1 is a crucial protein for autophagy initiation, which, together with PIK3C3 and PIK3R4, forms a protein complex Class III PI3K, and ultimately regulates the formation and maturation of autophagosomes [64–66]. Atg7 and LC3B are markers of autophagosome formation [67, 68]. LC3B is involved in the recruitment of p62 to autophagosomes [69], which is eventually degraded, and the protein level of p62 decreases [70]. The electron microscopy results of skeletal muscle mitochondria in the present study provide direct evidence of mitophagy in the DDP group manifesting increasing swollen, vacuolar damaged mitochondria, which were surrounded by the double membrane phagophore structure forming the autophagosomes. YFSJ treatment reduced the accidence of mitophagy and damage caused by DDP. In order to strengthen the evidence, mitophagy-related proteins were detected in this study. The results showed that YFSJ could reverse the expression level of DDP-induced mitochondrial autophagy-related proteins. Proper regulation of mitophagy is essential for normal cellular and physiological function [71]. However, excessive mitophagy can trigger cell death [72]. Skeletal muscle cells were also observed under the microscope. DDP induced severe damage such as twisted, broken, and irregular arrangements of muscle fibers and even apoptosis in the muscle tissue. Because in the process of mitochondrial autophagy, apoptotic factors (such as cyto. C) are released, which further activate Caspase-9 and Caspase-3 [73–77], ultimately transmitting the death signal to the downstream molecules, such as PARP [78], reducing the stability of DNA, to promote apoptosis. At the same time, ROS can also promote the release of cyto. C from mitochondria, leading to the occurrence of apoptosis [79]. In the present study, the results obtained from western blot revealed that the DDP group exhibited increased protein expression levels of cyto. C, c-Casp 9, c-Casp 3, and c-PARP in skeletal muscle tissues of mice, which were consistent with the results of the TUNEL staining assay. However, YFSJ can obviously reverse this phenomenon caused by DDP. To sum up, YFSJ treatment decreased oxidative stress, inhibited the activation of the AMPK/mTOR pathway, decreased protein expression levels of Beclin1 and other autophagy-related proteins, and attenuated the activation of cyto. C, c-Casp 9, and other apoptosis-related proteins in skeletal muscle tissues by DDP. These results revealed that YFSJ might reduce skeletal muscle apoptosis by alleviating oxidative stress-mediated mitophagy for the inhibitory effect on CRF.

Previous studies proved that promoting ROS production is the most critical pathway chemotherapy follows in cancer elimination [80, 81]. By increasing ROS concentrations (which may be associated with some components of YFSJ, such as Quercetin and Oleanolic acid [82, 83]), patients have high overall survival and a good prognosis as ROS overgeneration enhances DDP sensitivity and apoptosis induction [84]. In tumor cells, increased ROS content activates the JNK/p53 pathway, increasing Bax expression. Bax is a proapoptotic protein, and the increase of Bax will activate Casp3, and C-Casp3 will further promote PARP cleavage, which will reduce the stability of double-stranded DNA and eventually lead to apoptosis [85]. Meanwhile, the activation of p53 promotes the expression of DAPK1 [86], which further promotes the expression of Beclin1 [87], a key autophagy factor, and ultimately leads to autophagy in tumor cells. Similar results were observed in our study; YFSJ enhanced DDP sensitivity by promoting ROS and MDA production, decreasing the SOD concentrations, and activating the cell apoptosis and autophagy in the tumor tissues of mice. It was also found that YFSJ reduced the loss of body weight caused by DDP, ascended the serum levels of ALT, AST, and CREA, increased the spleen index, and prolonged the survival time of mice. In conclusion, YJSJ can specifically increase the ROS content of tumor cells and increase chemotherapy sensitivity. So, YFSJ can alleviate the hepatorenal toxicity and immunosuppression caused by DDP. Our results showed that YFSJ can inhibit tumor growth and improve the quality of life of patients.

In this study, we found an interesting phenomenon: YFSJ administration decreased the ROS concentrations in the muscle tissue but increased them in the tumor tissue. However, they were enhanced in whatever tissues after DDP treatment. In the tumor microenvironment of cancer cells, low to moderate levels of cellular ROS are produced to regulate cell signaling and promote cell proliferation [88], as one of the unique characteristics of cancer [89]. The persistent mild elevated level of ROS can provide metabolic reprogramming to deal with the stress induced by cancer therapies and even enhance tumor resistance [90]. However, unlike tumor tissue, skeletal muscle tissue typically has good blood oxygen content and is prone to produce excessive ROS under the cancer therapy's stress [91]. In addition to some of the components mentioned, as a traditional Chinese medicinal formula based on the concept of holism, YFSJ is often used to adjust the macroscopic state of the human body not only to resolve the masses to reduce the solid tumor but also tonify qi (vital energy) and improve the blood circulation. These effects of YFSJ can improve the nutritional status of the human body under DDP chemotherapy. Studies have confirmed that poor nutritional status can lead to excessive ROS production in mitochondria and activate autophagy [92, 93]. On the other hand, improving nutritional status may improve the body's ability to recognize tumors and break the relatively stable tumor microenvironment. Based on these functions, we assume that YFSJ might break the relatively stable hypoxic microenvironment of tumor tissue and promote ROS overgeneration to enhance DDP sensitivity. At the same time, improved blood circulation could accelerate the elimination of the excessive ROS produced by skeletal muscle cells due to stress. Further experiments will be carried out on this hypothesis in the future. This study was conducted only in vivo experiments but not in vitro verification. We will carry out in vitro verification experiments based on the results of this experiment in the future.

**Figure 6:**
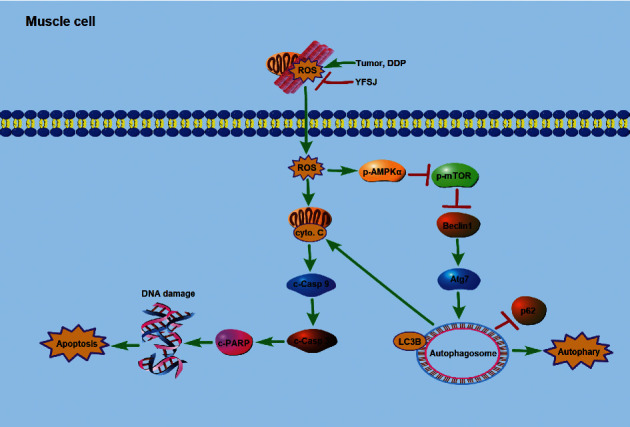
Schematic illustration of the potential underlying mechanism responsible for reducing skeletal muscle injury by YFSJ.

**Figure 7:**
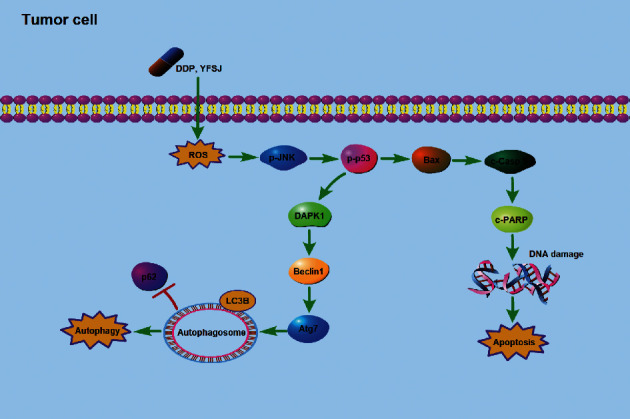
Schematic illustration of the potential underlying mechanism responsible for inhibiting tumor growth by YFSJ.

The hypothetical mechanism by which YFSJ alleviates CRF by reducing oxidative stress levels in skeletal muscle cells is shown in Figure 6. The hypothetical mechanism of YFSJ inhibiting tumor growth by increasing the ROS content of tumor cells to enhance chemotherapy sensitivity is shown in Figure 7. In conclusion, YFSJ specifically regulates ROS concentration in different tissues to reduce skeletal muscle injury and inhibit tumor growth and is directly and closely related to CRF treatment.

## 5. Conclusions

In this study, all results demonstrated that YFSJ could alleviate CRF by reducing mitophagy and apoptosis due to reducing oxidative stress of skeletal muscle. These results also displayed the effects of YFSJ on enhancing chemotherapy sensitivity, improving quality of life, and prolonging survival time in lung cancer mice who received DDP chemotherapy.

## Data Availability

The datasets used and/or analyzed during the current study are available from the corresponding author upon reasonable request.

## Supplementary Materials

Supplementary Materials Supplementary Figure 1: (a) The packaging and appearance of the YFSJ pill. (b) Representative figures of herbs in YFSJ. Supplementary Table 1: List of botanical, herbal, Chinese names of the corresponding herbs in YFSJ. Supplementary Figure 2: (a) Q-Orbitrap-LC/MS analysis base peak intensity chromatograms of YFSJ in positive mode. (b) Q-Orbitrap-LC/MS analysis base peak intensity chromatograms of YFSJ in negative mode. Supplementary Table 2: Identification of components of YFSJ by Q-Orbitrap-LC/MS analysis.
