# Supplementary material for: Yifei sanjie Pills Alleviate Chemotherapy-Related Fatigue by Reducing Skeletal Muscle Injury and Inhibiting Tumor Growth in Lung Cancer Mice
Source: Evid Based Complement Alternat Med. 2022 Aug 22;2022:2357616. doi: 10.1155/2022/2357616 (PMC9423986; doi:10.1155/2022/2357616)
Supplement: Supplementary Materials — Supplementary Figure 1: (a) The packaging and appearance of the YFSJ pill. (b) Representative figures of herbs in YFSJ. Supplementary Table 1: List of botanical, herbal, Chinese names of the corresponding herbs in YFSJ. Supplementary Figure 2: (a) Q-Orbitrap-LC/MS analysis base peak intensity chromatograms of YFSJ in positive mode. (b) Q-Orbitrap-LC/MS analysis base peak intensity chromatograms of YFSJ in negative mode. Supplementary Table 2: Identification of components of YFSJ by Q-Orbitrap-LC/MS analysis. [file 2357616.f1.docx]

### Picture and composition identification of *Yifei sanjie pill*

### Materials and reagents

### 2.1 Yifei sanjie pill (YFSJ) **preparation**

YFSJ was composed of eight herbs formed into pills preparation and was purchased from the First Affiliated Hospital of Guangzhou University of Chinese Medicine (Guangdong, China). YFSJ (8g /packet) was dissolved in 24 mL normal saline and the solution was promoted by eddy vibration with final concentration of 0.33 g/mL before using. Details of the herbal materials are listed in Supplementary Figure 1 and Supplementary Table 1.

(a)


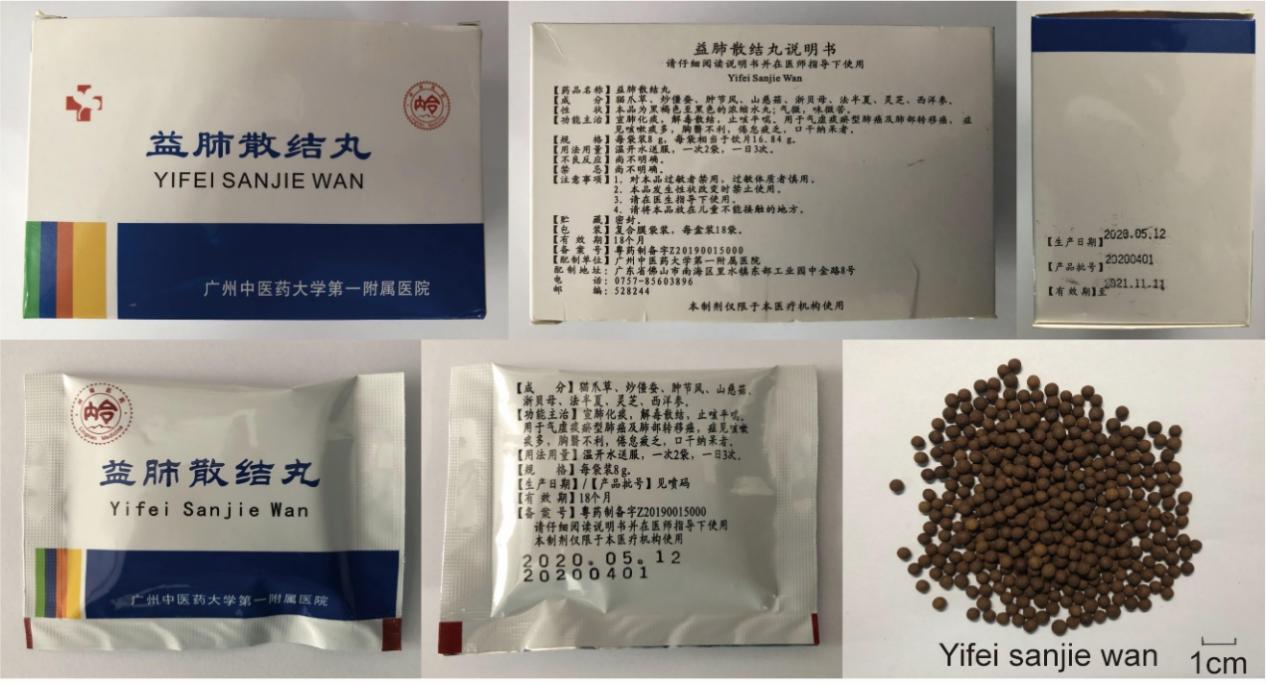


(b)


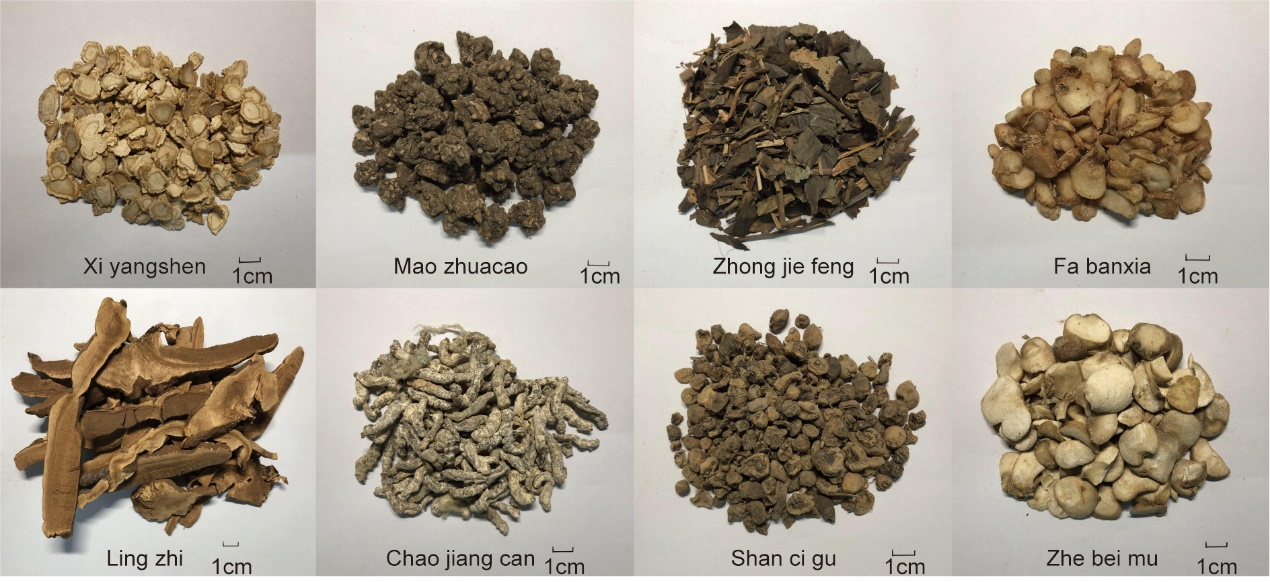


Supplementary Figure 1: (a). The packaging and appearance of the YFSJ pill. (b). Representative figures of herbs in YFSJ. Bar = 1 cm.

Supplementary Table 1: List of botanical, herbal, Chinese name of the corresponding herbs in YFSJ

| **Botanical name** | **Herbal name** | **Chinese name** |
| --- | --- | --- |
| *Panax quinquefolium* L. | Panacis quinquefolii radix | Xi yang shen |
| *Ranunculus terrnatus* Thunb. | Ranunculi ternati radix | Mao zhao cao |
| *Sarcandra glabra (Thunb.)* Nakai | Sarcandrae herba | Zhong jie feng |
| *Pinellia ternata (Thunb.)* Breit. | Pinelliae rhizoma praeparatum | Fa ban xia |
| *Ganoderma lucidum (Leyss. ex Fr.)* Karst. | Ganoderma | Ling zhi |
| *Bombyx mori Linnaeus* | Bombyx batryticatus | Chao jiang can |
| *Cremastra appendiculata (D.Don)* Makino | Cremastrae Pseudobulbus Pleiones Pseudobulbus | Shan ci gu |
| *Fritillaria thunbergii* Miq. | Fritillariae thunbergii bulbus | Zhe bei mu |

Chemical constituents of YFSJ were identified based on the Q-Orbitrap high resolution liquid/mass spectrometry(Q-Orbitrap-LC/MS). The data collected by high resolution liquid mass were processed by CD2.1 (Thermo Fisher) and then searched and compared in the database (MZCloud, MZVault, ChemSpider). The peak intensity chromatograms of chemical constituents in YFSJ were displayed in Supplementary Figure 2. The obtained compounds were cross-linked with the known traditional Chinese medicine components in YFSJ to screen out the possible compounds and listed in Supplementary Table 2.


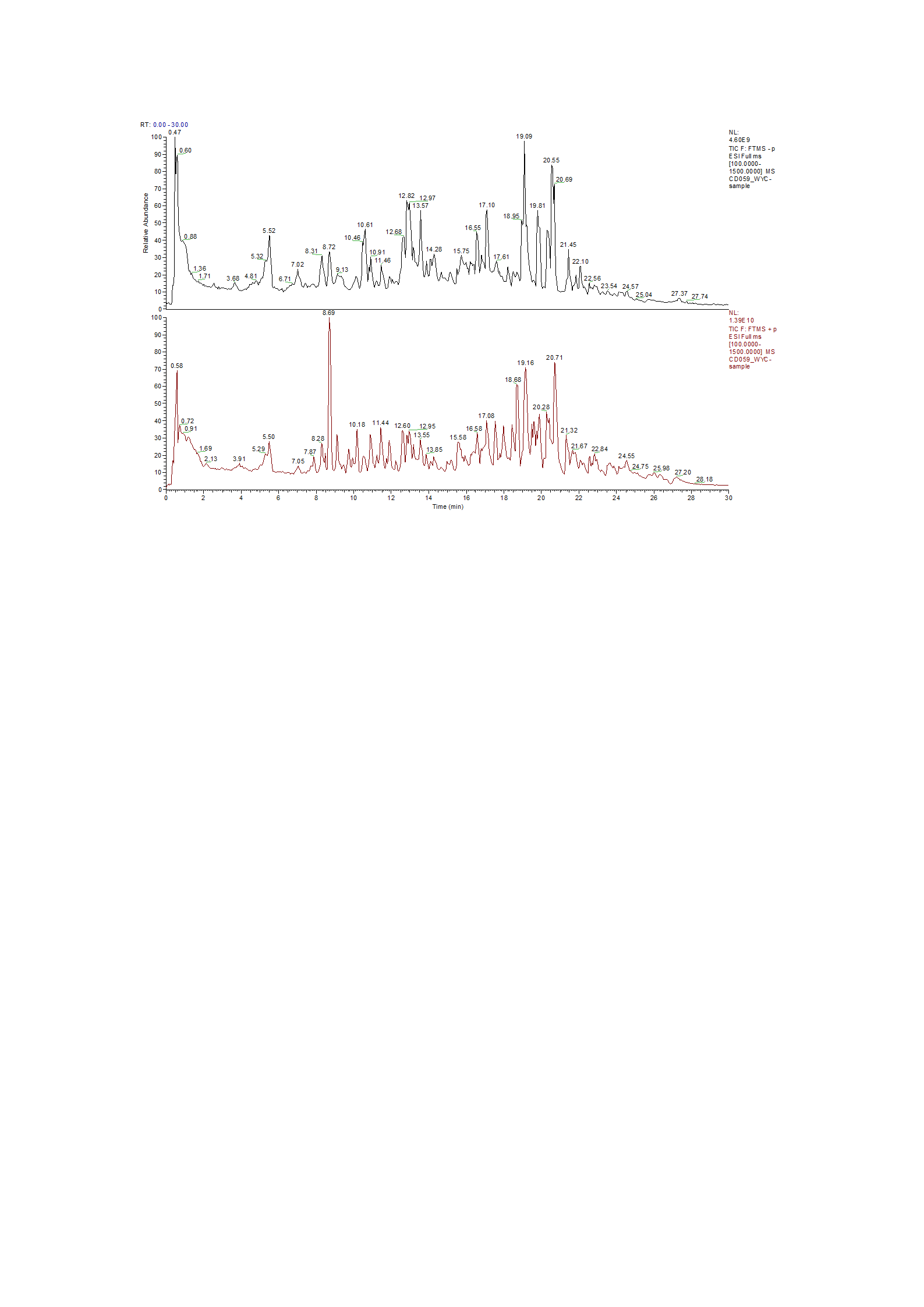


(b)

(a)

Supplementary Figure 2: (a). Q-Orbitrap-LC/MS analysis base peak intensity chromatograms of YFSJ in positive mode. (b). Q-Orbitrap-LC/MS analysis base peak intensity chromatograms of YFSJ in negative mode.

Supplementary Table 2: Identification of components of YFSJ by Q-Orbitrap-LC/MS analysis.

| **Herbal name** | **Name** | **mzCloud Best Match** | **FTMS** | **RT [min]** | **Area (Max.)** |
| --- | --- | --- | --- | --- | --- |
| Panacis quinquefolii radix (Xi yang shen) | Quercetin; | 100 | - | 11.81 | 1581360.615 |
|  | Oleanolic acid; | 98.4 | + | 19.455 | 8070888.778 |
|  | Kaempferol; | 93 | + | 11.244 | 1191795.934 |
|  | Ginsenoside Rd; | 88.7 | + | 16.796 | 18409307.65 |
|  | Arachidonic acid; | 82.6 | + | 17.908 | 136526017.2 |
|  | Ginsenoside Rg2; | 76.5 | + | 12.618 | 11029311.77 |
|  | Ginsenoside Rg3; | 76.3 | + | 15.585 | 18401380.52 |
|  |  |  |  |  |  |
| Mao zhua cao Ranunculi Ternati Radix (Mao zhao cao) | Azelaic acid; | 100 | - | 10.61 | 48679239.68 |
|  | Citric acid; | 99.5 | - | 0.899 | 217514214.8 |
|  | Suberic acid; | 91.4 | - | 8.929 | 3202415.179 |
|  | 9-Oxo-10(E),12(E)-octadecadienoic acid; | 80.7 | - | 14.195 | 47603393.24 |
|  | Succinic acid; | 41 | + | 1.036 | 9293142.354 |
|  |  |  |  |  |  |
| Sarcandrae Herba (Zhong jie feng) | Quercetin; | 100 | - | 11.81 | 1581360.615 |
|  | Scopoletin; | 88.4 | + | 8.394 | 7024076.063 |
|  | Succinic acid; | 41 | + | 1.036 | 9293142.354 |
|  |  |  |  |  |  |
| Pinelliae Rhizoma Praeparatum (Fa ban xia) | Gallic acid; | 99.7 | - | 1.729 | 2572726.334 |
|  | Palmitoleic acid; | 98.4 | + | 18.723 | 752284.0397 |
|  | Baicalein; | 89.1 | + | 13.044 | 3603146.76 |
|  | Trigonelline; | 85.8 | + | 0.532 | 7614756.783 |
|  | Hypoxanthine; | 85.3 | + | 0.859 | 9575621.515 |
|  | 9-Oxo-10(E),12(E)-octadecadienoic acid | 80.7 | - | 14.195 | 47603393.24 |
|  | Succinic acid | 41 | + | 1.036 | 9293142.354 |
|  |  |  |  |  |  |
| Ganoderma (Ling zhi) | D-Glucosamine | 98.4 | + | 0.382 | 6869563.784 |
|  | Nicotinic acid | 93.4 | + | 0.716 | 28051891.18 |
|  | 9-Oxo-10(E),12(E)-octadecadienoic acid | 80.7 | - | 14.195 | 47603393.24 |
|  |  |  |  |  |  |
| Bombyx batryticatus (Chao jiang can) | Beauvericin; | 96.2 | + | 18.021 | 27722451.99 |
|  |  |  |  |  |  |
| Cremastrae Pseudobulbus Pleiones Pseudobulbus (Shan ci gu) | Methyl cinnamate; | 64.2 | - | 9.985 | 2045257.027 |
|  | Succinic acid; | 41 | + | 1.036 | 9293142.354 |
|  |  |  |  |  |  |
| Fritillariae Thunbergii Bulbus (Zhe bei mu) | Succinic acid; | 41 | + | 1.036 | 9293142.354 |
